# Multi-Level Evolution for Robotic Design

**DOI:** 10.3389/frobt.2021.684304

**Published:** 2021-06-29

**Authors:** Shelvin Chand, David Howard

**Affiliations:** Commonwealth Scientific and Industrial Research Organisation, Brisbane, QLD, Australia

**Keywords:** evolutionary robotics, map elites, optimization, shape grammar, evolutionary algorithms

## Abstract

Multi-level evolution (MLE) is a novel robotic design paradigm which decomposes the design problem into layered sub-tasks that involve concurrent search for appropriate materials, component geometry and overall morphology. This has a number of advantages, mainly in terms of quality and scalability. In this paper, we present a hierarchical approach to robotic design based on the MLE architecture. The design problem involves finding a robotic design which can be used to perform a specific locomotion task. At the materials layer, we put together a simple collection of materials which are represented by combinations of mechanical properties such as friction and restitution. At the components layer we combine these materials with geometric design to form robot limbs. Finally, at the robot layer we introduce these evolved limbs into robotic body-plans and learn control policies to form complete robots. Quality-diversity algorithms at each level allow for the discovery of a wide variety of reusable elements. The results strongly support the initial claims for the benefits of MLE, allowing for the discovery of designs that would otherwise be difficult to achieve with conventional design paradigms.

## 1 Introduction

The Multi Level Evolution (MLE) architecture, proposed by Howard et al. (2019), is a three-layer framework for discovering new robotic designs. Each layer deals with a different aspect, starting with the lowest tier where materials are discovered, followed by the second layer where the components are created by considering a combination of materials and geometry, and finally leading to the third layer where components are combined into specific body plans to form complete robots. The framework relies heavily on illumination/quality-diversity algorithms which search for high performing solutions across multiple diverse feature dimensions.

MLE has a number of key potential advantages. Firstly, it has the ability to automatically consider a huge range of geometric and material combination possibilities. Secondly, it is highly scalable since all three layers can perform search independently and in parallel. Furthermore, the architecture is “self-optimising” in that the longer it runs, the more options are discovered for use by the design algorithms. Finally, it also allows for re-usability due to the separation of search processes between the layers. Components discovered in one search process can be re-used across design tasks to form complete robots. MLE search could target a myriad of impact areas, including underwater, mining, industrial inspection, and remote surveying. Despite these potential benefits, Howard et al. (2019) did not present any algorithmic implementations or experimental results in their study to verify their claims.

In this paper we present the first instantiation of the MLE architecture, a hierarchical robotic design approach with three layers. Instantitation in this context refers to an actual algorithmic implementation of the MLE framework combined with an evaluation and analysis of its performance on an evolutionary robotics task. We focus on the design of multi-legged robots. At the lowest layer, we provide a library of materials based on combinations of mechanical properties. At the components layer, we design robotic limbs by combining the materials with complex geometry, generated using a shape grammar. Finally, we put together the evolved legs into a complete body plan and optimize the controllers for the desired movement. We test our evolved robots in two different environments and demonstrate the benefits of our hierarchical approach. Our approach harnesses MAP-Elites (Vassiliades et al., 2018) to generate a diversity of designs at each layer, given its noted success in similar scenarios. In particular, the key intended contributions of this paper are as follows:

• The first instantiation of an MLE system: a three layer hierarchical approach to robotic design that explicitly considers materials, components and complete body-plans.
• A new representation for robot legs based on point clouds and shape grammar, allowing for a wide variety of complex designs.
• A detailed analysis of the simulation results demonstrating the envisioned benefits of MLE as detailed by Howard et al. (2019). The self-optimising nature of the overall architecture is shown. Cross environment applicability of the evolved designs is demonstrated by transferring between two different environments, namely a high friction surface and a low friction (icy) surface.

The rest of the paper is organised as follows. Section 2 provides background on the relevant literature. Section 3 gives details on the proposed hierarchical approach for evolving robots. Section 4 provides experimental details and analysis on the results. Finally, Section 5 summarizes the findings of the paper and highlights some future research directions.

## 2 Background

### 2.1 Evolutionary Robotics

Evolutionary Robotics (Cliff et al., 1993; Nolfi et al., 1994; Doncieux et al., 2015) as a field of research focuses on applying principles of natural evolution (i.e., selection, recombination, etc.) to the design of robots whose behaviour is typically elicited through embodied cognition. This may involve optimizing the controller (Cully and Mouret, 2016), the physical design (Collins et al., 2019) or both (Auerbach and Bongard, 2011). Optimization focusing on body-brain evolution remains complex since small changes in body design can lead to the need for major changes in the control strategy, and vice versa.

The seminal work of Sims (1994) evolved complex robots represented by directed graphs, where the nodes and edges represented a number of aspects ranging from dimensions of body-parts to controller specifications. This was followed by a number of studies (Mautner and Belew, 2000; Hornby and Pollack, 2001; Auerbach and Bongard, 2010, Auerbach and Bongard, 2011) which also focused on body-brain evolution. The obvious advantage of this approach is that it automates the entire design process instead of relying on human engineers, whose design processes are (comparatively) limited in scope and may include bias (Auerbach and Bongard, 2011). The entire design space can be explored leading to sometimes unconventional, yet optimal or near optimal results. This approach can be further combined with rapid prototyping to create real world robots (Lipson and Pollack, 2000). Recent studies on body-brain evolution focus on the effect of the environment on design complexities (Auerbach and Bongard, 2014; Miras and Eiben, 2019), similar to the environmental setup of the experiments used in this article. MLE is envisioned to offer a solution to specific problems encountered in evolutionary robotics, particularly regarding re-use of designs (as already-discovered components may be tweaked or re-used for various applications without re-running the entire evolutionary process) and the emergence of complexity (as materials, components, and robot evolution are separated and can occur independently).

### 2.2 Modular Robotics

Modular robotics deals with constructing robots by exploring the arrangement of reusable components (Hornby et al., 2001). A number of studies have focused on evolving modular robots. Hornby et al. (2001) evolved 2D modular robots using an L-Systems encoding for the morphology. The robots were built using bars and actuators as the basic re-usable building blocks. Van Diepen and Shea (2019) used shape grammar to evolve soft robots which were put together based on combinations of static hand-designed soft 3D actuated shapes. Miras and Eiben (2019) used L-Systems to evolve morphologies for modular robots built using robogen components. The existing literature on modular robotics mostly focuses on evolving morphologies or controllers for robots built using hand-designed modular components. MLE can be considered a modular robotic system that also automatically designs the components themselves.

### 2.3 Quality-Diversity Algorithms

Illumination algorithms are different from traditional evolutionary algorithms in the sense that they focus on achieving quality through diversity. These algorithms typically return a collection of solution that cover the feature space. Novelty search (Lehman and Stanley, 2008; Lehman and Stanley, 2011a) is an illumination algorithm which rewards novelty and diversity. The algorithm replaces the fitness function with a novelty metric which creates pressure towards further exploration of the search space and generation of solutions which exhibit novel behavior with respect to solutions within the current population and those previously encountered and stored within an archive. One limitation of novelty search is that since there is no drive towards functionality, this may result in solutions which are diverse in behavior or characteristics but not functional and hence of limited use. To solve this problem the authors extend novelty search by combining it with the concept of local competition (NSLC) (Lehman and Stanley, 2011b) within a multi-objective paradigm. Local competition creates a performance pressure within niches encouraging better, more functional solutions within each niche. In this way, novelty search encourages the discovery of new niches while local competition allows for performance improvement within those niches.

Another type of illumination algorithm is MAP-Elites (Mouret and Clune, 2015). For each solution *x*, MAP-Elites maintains an objective value f(x) and a *N*-dimensional feature vector characterising the solution across the various features of interest. For this reason a feature or behavior function b(x) also needs to be defined which computes *x*’s value across the *N* feature dimensions. The algorithm starts by generating a set of random solutions and computing their objective and feature values. These solutions are then placed into the appropriate cells in the feature map. In case of a cell having more than one solution, only the highest performing solution (in terms of objective value) is retained. From here on, the solutions within the feature map undergo recombination to generate new solutions which are evaluated and then assigned to appropriate locations in the feature map. This process is repeated until termination.

This algorithm has been successfully used for tackling problems with 2–6 feature dimensions (Cully et al., 2015). Extending beyond 6 feature dimensions is a challenging task since the feature cells increase exponentially leading to a reduction in selection pressure. To counter this, Vassiliades et al. (2018) proposed Centroidal Voronoi Tessellations (CVT) MAP-Elites which divides the feature map into desired number of maximally spread niches. CVT MAP-Elites behaves the same as the original MAP-Elites with the only exception being the way in which the feature map is structured. Essentially what CVT does is partition the feature space into regions with each having a centroid. The closest centroid to a given solution determines the region into which it will be placed. MAP-Elites and its variants have been used in a number of different applications, including robotics (Nordmoen et al., 2020), video games (Fontaine et al., 2019) and routing logistics (Urquhart and Hart, 2018).

Multi-objective optimization based approaches (Clune et al., 2013; Brandão et al., 2020) have also been used in the quality-diversity space. The algorithm in (Brandão et al., 2020) for example achieves diversity in the design space as a consequence of seeking diversity within the trade-off between objectives. In this work, we use CVT-MAP-Elites for maintaining solution libraries, however, the quality-diversity literature presents many different options to consider for future research.

### 2.4 Shape Grammar

Shape grammar is a generative design procedure which uses shape primitives encoded into design rules to generate more complex shapes. This was initially proposed by Stiny and Gips (1971) and has since been used in a number of different fields including architectural design (Downing and Flemming, 1981), computer graphics applications (Stiny, 1982) and robotic design (Van Diepen and Shea, 2019). Robots designed using shape grammar are usually expressed as a sequence of rules where the rules express the physical arrangement and structure of the robot. Van Diepen and Shea (2019) presented a shape grammar based approach for the design of soft robots. Their manually designed rules focused on small building blocks and their activation patterns, offsets, material properties, etc. Zhao et al. (2020) proposed a graph grammar based approach for automated design of robots to traverse given terrains. At the core of the grammar are a set of pre-defined building blocks for the robot expressed in the form of shapes, sizes, connection angles, etc. The grammar itself is manually designed to ensure fabrication feasibility. In both the above cases the grammar has been manually designed by human experts. However, due to the obvious limitations of what a human expert can consider in a limited time, this approach significantly limits the design exploration.

## 3 Methodology

### 3.1 Overall Architecture

The method presented in this paper uses a hierarchical approach to robot design and is the first instantiation of the MLE Architecture. The bottom layer is the materials layer, which consists of a set materials with pre-generated mechanical properties. Above that is the components layer where robot legs are designed using a combination of point clouds and shape grammar. The geometric leg shapes are also assigned materials which are picked from the materials layer. Finally, the robot layer combines the legs into body plans to form complete robots which can perform locomotion tasks. Optimization at the components and robot layer are done using CVT-MAP-Elites (Vassiliades et al., 2018).

### 3.2 Materials Layer

The materials layer is composed of a set of pre-loaded materials. In reality materials are characterised by a number of complex characteristics. However, for simplicity, in this research we consider a material to be characterised by two main properties, namely coefficient of friction and coefficient of restitution. For both we consider four discrete values, [0.25, 0.50, 0.75, 1.0]. As a result we end up with 16 different combinations of the two properties and hence 16 different materials. In future research, it would be worth considering more complex characteristics, and potentially evolving materials (Howard et al., 2019).

### 3.3 Components Layer

#### 3.3.1 Representation

A solution in the components layer is made up of various elements with shape grammar at the core. First, we have a set of base shapes (irregular polygons). Each of these shapes are constructed using a point cloud. For each point cloud we generate 10 random points and perform a convex hull to get the final form of the irregular polygon. This is repeated for however many base shapes that are needed. The point cloud representation is inspired by the work of Muehlbauer et al. (2017) in which they used point clouds to define and evolve 3D architectural shapes. Each of the base shapes are then assigned a material from the materials archive and two edges are identified (randomly) for in-ward and out-ward connection with other shapes. Each of the base shapes are also assigned an ID from 1 to *n* where *n* is the total number of base shapes.

**FIGURE 1:**
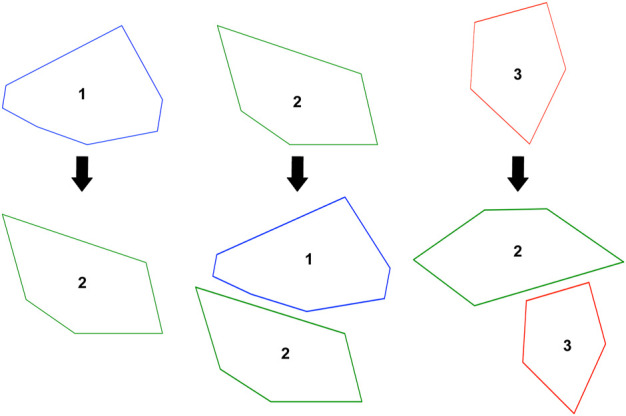
An example of a randomly generated grammar with three base shapes. Arrows represent the transformation eg., each occurrence of shape three is replaced with a combination of shapes 2 and 3 as given by the rule. Orientations have been changed in some cases depending on connecting edges.

**FIGURE 2:**
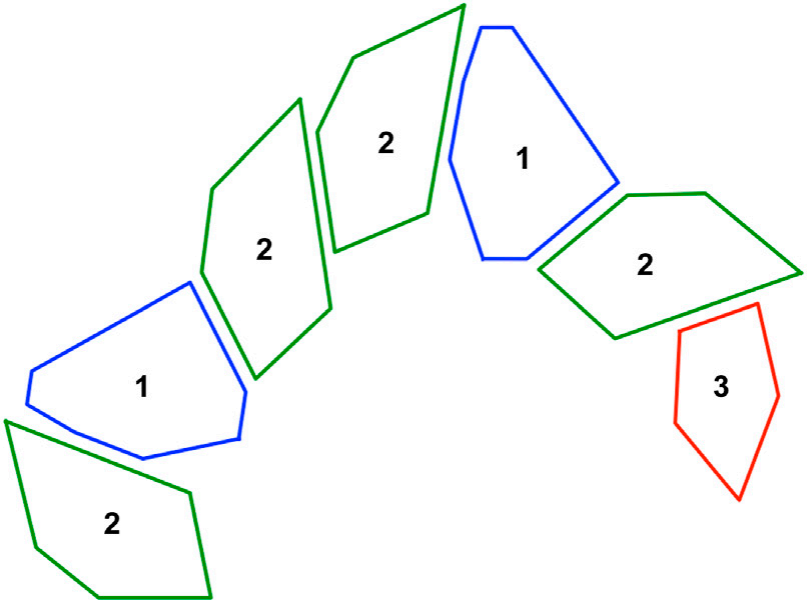
The resulting outcome from using the Grammar in Figure 1 and a starting point (axiom) = 2 with three iterations. Each shape is connected to the next one using a fixed joint or revolute joint.

Next, a set of rules are generated based on these base shapes. These rules will be iteratively applied to form a complete leg from these base shapes. A transformation rule is generated for each of the base shapes in the form of A → [B] or A → [B,C] where A is the *i*th base shape and B and C are base shapes chosen from the set of all base shapes. This essentially means that in every iteration, all occurrences of A will be replaced with [B,C] where shapes B and C are connected to each other (in the case of the second rule). B and C can be different from each other, same or even equal to A. Next an axiom is determined by randomly picking a base shape ID which will start the leg shape generation process. In every iteration, the rules are applied by replacing elements from the left hand side (LHS) of the rule with those from the right hand side (RHS). The two different types of rule RHS allow us to vary the length of the leg. Example of a randomly generated shape grammar is given in Figures 1, 2. These designs are then converted into 3D meshes. This is done by adding a constant height to each of the shapes (building blocks) within a leg design and converting it into a 3D trimesh.

This representation has a number of advantages. Firstly, it is fairly simple and easy to understand and implement. Furthermore, fairly complex shapes and structures can be formed by combining a relatively small set of point-cloud meshes. The angles at which these meshes are connected allow for complex movement which would be difficult to achieve when using standardized or regular shapes such as cubes (Lassabe et al., 2007) or spheres (Auerbach and Bongard, 2011).

#### 3.3.2 Recombination

The evolutionary process at the components layer uses four mutation operators. The first operation replaces a randomly selected base shape and all its properties (connection edges) with a new randomly generated shape. The second operation replaces a randomly selected rule with another randomly generated rule. The third operations replaces the current axiom with another one, selected randomly from the set of base shapes. Finally, the last operation selects a base shape at random and replaces the assigned material with a new one from the materials archive. The choice of operation is determined randomly.

#### 3.3.3 Fitness and Features

The fitness function used here is also taken from (Muehlbauer et al., 2017). This function (Eq. 1) averages the volume to surface area ratio across all *m* individual meshes (building blocks) that make up a leg. It ensures that the evolved shapes that make up a leg are compact, by maintaining an appropriate proportion between volume and surface area. Essentially it tries to avoid really long flat shapes which are not ideal from a design perspective. The features used by MAP-Elites are mean friction across the *m* building blocks, mean restitution across the *m* building blocks, leg size and leg complexity.

**FIGURE 3:**
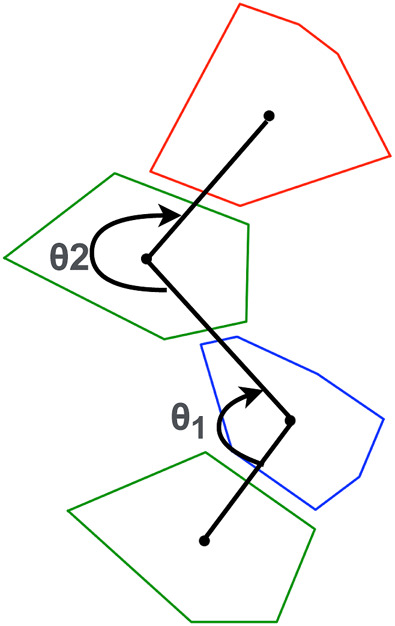
A sample leg design. The average of the two angles determines the complexity of the leg.

Leg complexity is measured as a function of the angles formed between the centroids of the shapes that make up the leg. To calculate this we consider a window of three shapes and keep moving this window till we reach the end of the leg. These angular values are averaged and assigned as leg complexity. The term “complexity” here is used loosely since the measure itself isn’t linearly indicative of complex designs. For example values close to 180 will indicate straight long legs which would be considered the most simple form of design. But as we move away from 180 we get to see more variation and a bit more complexity in terms of unorthodox overall leg shapes and structures. If a leg is composed of only 1 or 2 shapes, we assign default value of 180. This calculation is illustrated in Figure 3. Leg length is measured as the length of the line connecting all the centroids of the different shapes that form the leg.

$$Fitness=\left(\sum_{i=1}^{m}\frac{Volume_{i}}{SurfaceArea_{i}}\right)/m$$ (1)

### 3.4 Robot Layer

#### 3.4.1 Representation

**FIGURE 4:**
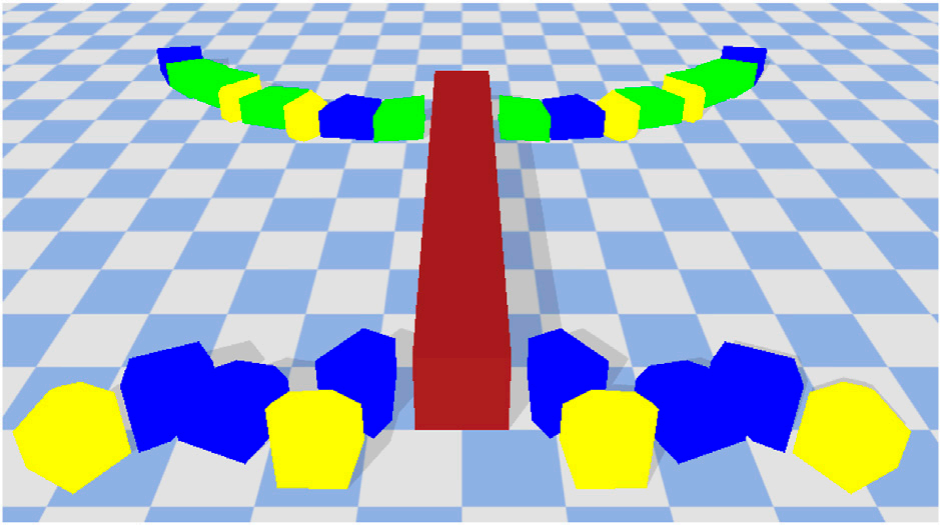
An example of a complete robot. The colors denote friction values. Green = 0.25, Red = 0.5, Blue = 0.75, and Yellow = 1.0. The cross-section (maroon) is always given a default friction value of 0.5. The robot initially starts in a flat position but can move in several ways including crawling, jumping, walking and so on.

A robot is defined by a body plan and a controller. The body-plan consists of a rectangular cross-section and a set of legs. We limit the number of legs between 2 and 6. The length of the cross-section is determined based on the number of legs (2 legs = 1 unit length, four legs = 8 unit length, 6 legs = 16 units length). The legs themselves are picked from the component layer archive and applied to the body-plan in a symmetrical manner (left and right). The leg designs in the components layer are not actuated. Hence, here we also evolve the joint movement-type between each of the individual blocks in a leg as well as between the leg and the cross-section. Each joint can be of type revolute or fixed. The revolute joints can allow movement in either the x or *z* direction. An example of a complete robot is given in Figure 4.

The controller is defined by a sinusoidal wave (Veenstra and Glette, 2020) as given in Eq. 2. *A* represents the amplitude, *ω* represents the frequency, *φ* represents the phase and $D_{j}$ represents the joint offset for joint *j* on the right hand side of the robot. Since the legs are attached in a symmetrical manner, we only have to compute offsets for one side and these can be applied to both the corresponding joints on each side. All variables are bounded between -1 and 1 and optimized using a 1 + 1 EA (Droste et al., 1998). The total number of variables depend on the size of the robot and number of non-fixed joints. The output represents the desired change in joint position for joint *j* at time step *t*. This value is scaled to the appropriate movement range given in Table 1.

$$y{\left(t\right)}_{j}=A\sin\left(\omega t+\varphi \right)+D_{j}$$ (2)

**TABLE 1:** Parameter choices.

Parameter	Value
MAP-Elites	
Batch size	100
Generations [Robot layer]	1,000
Generations [Component layer]	20, 100, 1,000
Initial Batch size	1,000
Niches	1,000
Initial proportion of filled niches	0.1
Shape Grammar	
Point Cloud size	10
Number of base shapes	3
Grammar iterations	3
Simulation Environment	
Time (steps)	30 sec (7,200 steps)
Ground friction	0.05, 0.9
Ground Restitution	0.05, 1.0
Robot Body-Plan	
Number of legs	2, 4, 6
Joint upper limit (radians)	0.2
Joint lower limit (radians)	−0.2
Joint delta movement (radians)	[−0.05, 0.05]
Controller Evolution via 1+1 EA	
Trials (runs)	1
Iterations	20
Variable limits	[−1, 1]

#### 3.4.2 Recombination

The evolutionary process at the robot layer uses four mutation operators. First operation chooses a leg at random and replaces it with another one from the components archive. This change is applied symmetrically. The second operation mutates the joint type from the options given above. The third operation mutates the number of legs and hence legs are randomly added or deleted to reflect the new size of the robot. Finally, the last option keeps the design as it is and re-optimizes the controller. The choice of operation is determined randomly.

#### 3.4.3 Fitness and Features

The goal of this design process was to discover robots which can move seamlessly across a given environment/surface. A 1+1 EA (Droste et al., 1998) was used to search for control strategies which would maximise this movement. For each evaluation of a candidate controller, the robot designs were loaded into the simulator and the movement (based on the controller) were recorded. In the end, the candidate controller resulting in the greatest movement across the desired surface was picked.

The fitness of the controller, which is the distance covered in the *x*-direction, was taken to be the fitness of the overall robot. The features used by MAP-Elites are the average friction coefficient, restitution coefficient and leg size across all legs. If at any point the robots step outside the given y-boundary or changes orientation beyond an allowed threshold, the simulation is stopped and that particular candidate controller is assigned fitness of 0. This is done to avoid situations where the robot starts moving diagonally or simply tries to rotate around to gain distance.

**FIGURE 5:**
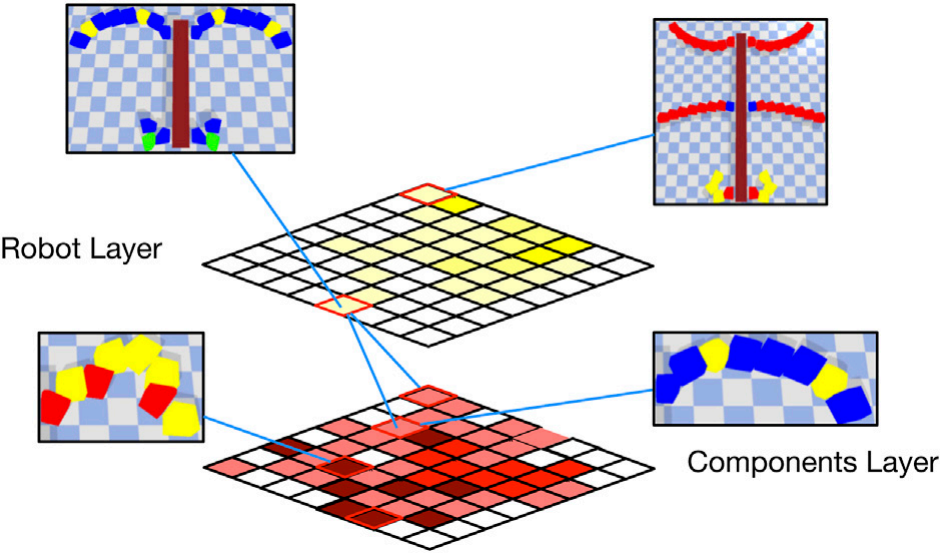
A conceptual diagram for the MLE instantiation illustrating the robot and components layer.

A summary of the overall structure is give in Figure 5 and in Algorithm 1. One of the obvious benefits of this layered approach is the modularity, in the sense that the materials archive and the components archive can be re-used. For example, one would only have to generate the components library once and this can be re-used for designing robots across different tasks. This would save time that would otherwise be spent on re-running the optimization algorithm.

**Algorithm 1:** Methodology

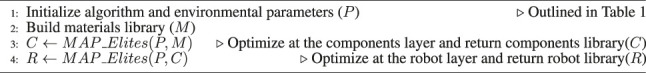

## 4 Experiments and Results

### 4.1 Experimental Setup

All simulations were run using the robotic simulator PyBullet. Robot designs were loaded into the simulator and the controller outputs were used to control the movement. Two different environments were used. First one is a high friction flat terrain characterised by friction = 0.9 and restitution = 1.0. The second one is a icy flat surface with fiction = 0.05 and restitution = 0.05. Friction on the robot and the environment only applies in terms of lateral friction. All self-collision was turned off for simplicity. 10 independent runs were done for each environment. As mentioned earlier, both the components and robot layer are modelled using CVT-MAP-Elites.

In our experiments we focus on two key aspects. First is the self-optimizing nature of the MLE architecture. This is done by having different termination conditions for the components layer. We consider 20 generations, 100 generations and 1,000 generations as the three termination conditions. We then try to evaluate the quality of solutions that are produced in the robot layer when working with component archives which have been formed in runs with these three termination conditions. Next we try to assess the cross environment applicability of the evolved robots. We take robots which have been trained on the high friction surface and evaluate them on the icy surface. The idea is to identify the types of designs that perform well on both environments while only being explicitly trained on one.

MAP-Elites at the robot layer is always run for 1,000 generations in all cases. Both the components and robot layer are divided into 1,000 niches. All other parameter choices are summarized in Table 1. The code for this research is available online^1^. All the experiments were performed on the CSIRO HPC cluster. Two different environments and three different termination conditions at the components layer resulted in six different setups. With 10 runs for each setup, we had a total of 60 evolutionary runs executed in parallel. Each run was on a separate node with 20 cores each. A typical evolutionary run took about 5 days.

### 4.2 Results

Through the experimental results, we aim to show the aforementioned benefits of the MLE architecture, namely, self-optimization and cross-environment applicability through the generation of re-usable components.

#### 4.2.1 Self-Optimization

**FIGURE 6:**
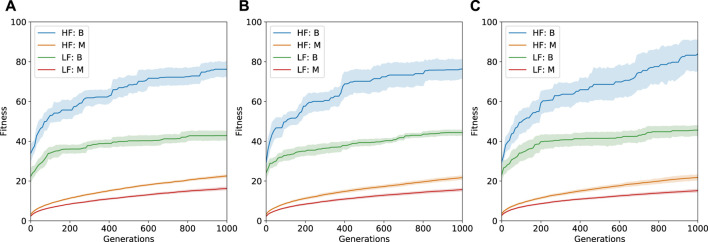
Performance across 10 runs at the robot layer with varying levels of computational budget at the components layer (A: 20 Generations, B: 100 Generations, C: 1,000 Generations). The plots show median and best performance across the two environments with 95% confidence interval. HF = High Friction Environment, LF = Low Friction Environment, B = Best, M = Median. Fitness is the distance covered in the *x*-direction.

Figure 6 shows the best and median performance for the two environments with varying levels of computational budget allocations for the components layer. The results indicate that locomotion across a high friction surface is relatively easier in comparison to a low friction surface. Robots evolved on high friction surface exhibited higher fitness values in both the best and median cases. Low friction surfaces tend to be more difficult and require much more intelligent control strategies to be able to navigate.

The MLE architecture is self-optimizing in nature and hence the accuracy/efficacy of the model improves as time passes (represented here with an increase in computation budget). In our experiments we observe an increase in quality of solutions at the robot layer as more and more generations of computational budget is assigned to the components layer. This is much more apparent for the robots evolved specifically for the high friction surface. A lower computational budget means that the coverage of the components library will be less. This would result in fewer options for the robot layer to utilize in terms of building complete robots. Having to work with a smaller components library can be restrictive and will affect the overall quality of the solutions.

Figure 6C depicts the case where the components layer was allocated 1,000 generations. For the high-friction surface, the benefits are very clear, especially in the case of the best solutions found across generations. Slight improvements are also visible for the low friction surface. Based on this, for the rest of the paper we focus on results obtained using 1,000 generations at both the components and robot layer.

#### 4.2.2 Coverage and Evolved Behavior

**FIGURE 7:**
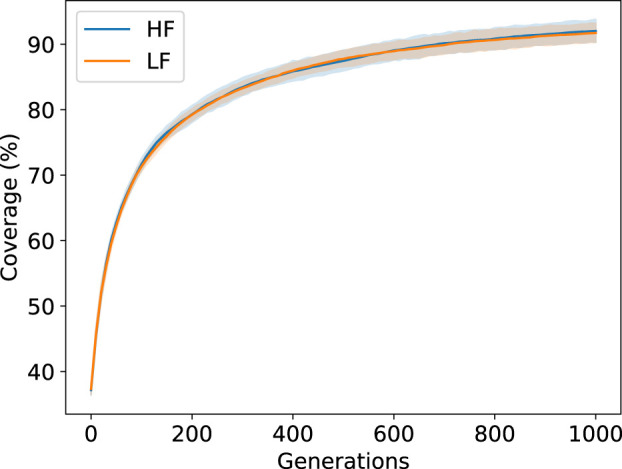
MAP-Elites archive coverage for the two different environments across the 10 runs.

For a typical run the coverage (percentage of occupied cells) of the feature map (Figure 7) at the robot layer is around 90 percent. This coverage would be sufficient for providing engineers or decision makers with a wide array of options to choose from.

**FIGURE 8:**
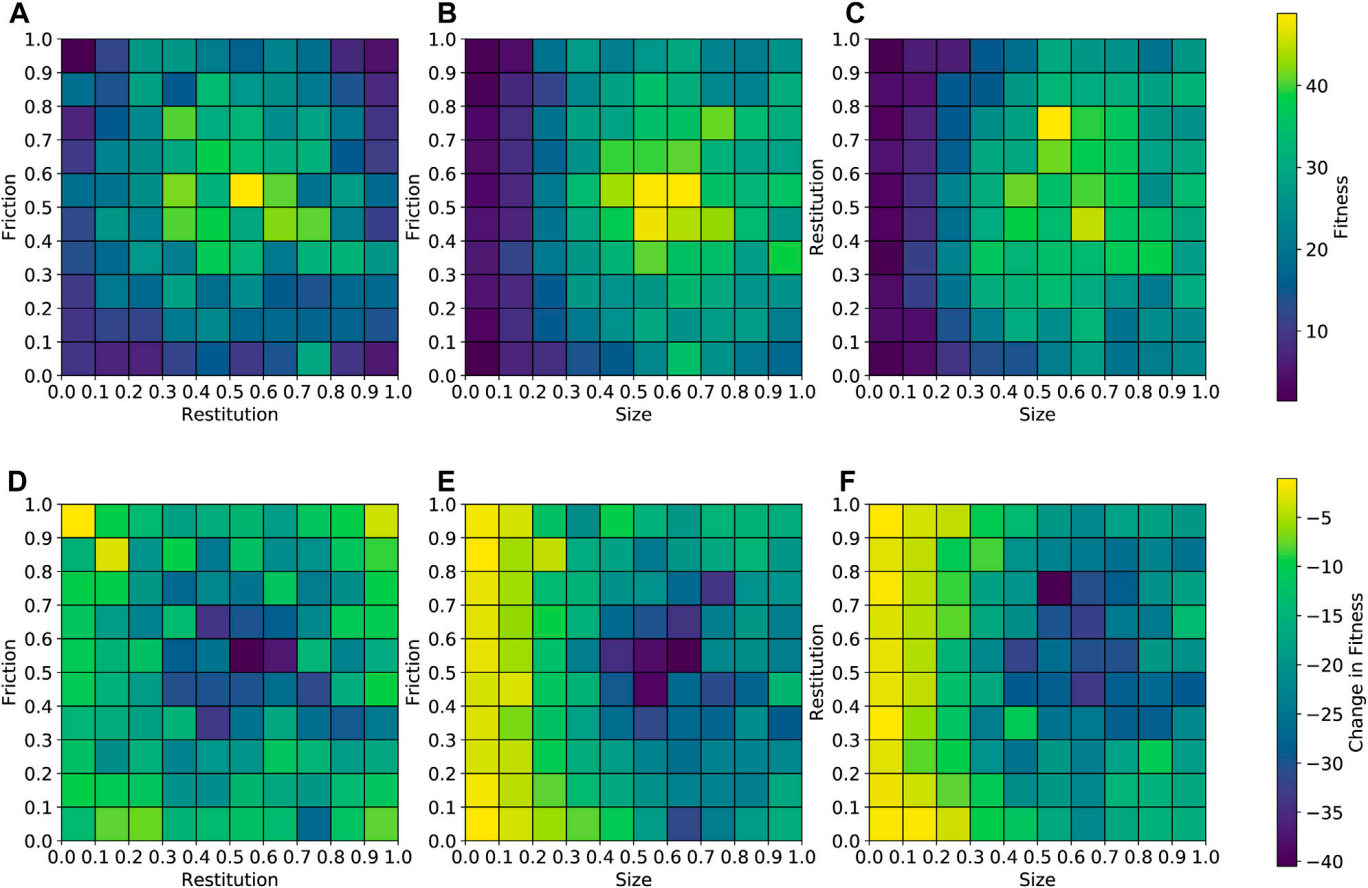
Pair-wise feature-fitness plots for the high friction surface. Plots A, B, and C show an aggregated view of how performance varies across the feature space. Plots D, E, and F show the effect of transferring the same solutions on to the low friction surface. Fitness refers to the distance covered by the robot in the *x*-direction. All feature values have been normalized between 0 and 1.

Figure 8A–C shows pair-wise feature-fitness plots for a typical run on the high-friction environment. CVT-MAP-Elites divides the feature space into maximally spread niches which are not typically in the form of perfectly sized boxes or cubes. Instead, each niche can have a different shape or form depending on the location of the feature centroids. Hence, only for visualization purposes, we have discretized the feature space into a grid with equi-sized boxes/squares. This not only makes it easier to visualize, but also helps highlight any inherent trends. We have aggregated the solutions (from the 1,000 niches) into 100 discrete boxes. This means that each box will represent an average of several solutions which fall into that discrete combination of features. It must be noted that the data is normalized between 0 and 1 so a friction value of 0 does not refer to 0 friction but rather the minimum value of 0.25. The same applies to the two other features.

Figure 8A–C reveal some interesting trends. Firstly, robots with shorter legs tend to perform poorly. This is obvious as longer legs allow for more range of motion including the ability to lift the cross-section off the floor which would minimize friction force from the floor. The effect of the material properties is a bit less straightforward. There seems to be a sweet-spot in the middle. The controller optimization strategy plays an important role in this. Regardless of the material properties, it will try to find controller parameters that can get maximum movement from the robot. However, material properties on the extremes can be a bit of a problem. For example, a robot with very low frictional properties would not be able to make a proper grip with the environment and will have to rely solely on sliding/crawling movements while robots with very high frictional properties may be held back due to the strong resistance from the environment. Having an average friction close to the middle (not too high, not too low) seems to allow the robots to gain maximum movement. It must be noted that the material properties shown in the plot are an average across all the blocks in all the legs. Hence a robot may have a combination of high and low friction blocks. Robots located in the middle region can benefit from both types of blocks and combine lift and forward thrusting movements with sliding and crawling. Alternatively the controller optimization may also try to cancel out the effects of unwanted blocks by creating a lifting motion to avoid interaction with the environment. However, this places additional burden on the controller optimization process which may not always yield the desired outcome.

Movements observed here are different from a traditional hexapod since the different pairs of legs on a robot can be of different sizes, complexity and material composition in comparison to other pairs.

#### 4.2.3 Cross Environment Performance

It is important to analyze the robustness of the evolved robots, i.e., how well do robots evolved specifically for high friction surfaces perform on low friction surfaces. Plots D, E, and F in Figure 8 illustrate the change in fitness when solutions evolved for the high friction surface are transferred to the low friction surface. It is important to note that the reason we have chosen to focus on high friction to low friction environmental transfer as opposed to vice-versa is because we consider the high friction environment to be slightly easier. This is visible from the fitness values given in Figure 6. Hence, we feel that it is of greater interest to see how solutions transfer from a simpler to a more complex or difficult environment.

First thing to note is that the loss in fitness is negative in nearly all cases since the low friction environment is expected to be more challenging. Secondly, we see that solutions with shorter legs continue to perform badly when transferred onto a different environment. As mentioned earlier, the shorter legs severely limit the range of motion and this is a factor that will limit movement regardless of the type of environment. Next we see that solutions in the mid-friction/restitution region which performed really well on the high friction surface, tend to transfer poorly onto the low fiction environment. It may be because of material properties or control behaviours that are too specific to the high friction surface. The solutions that tend to perform the best are those which are directly surrounding the “sweet-spot” region. These are solutions that perform reasonably well on both environments.

**FIGURE 9:**
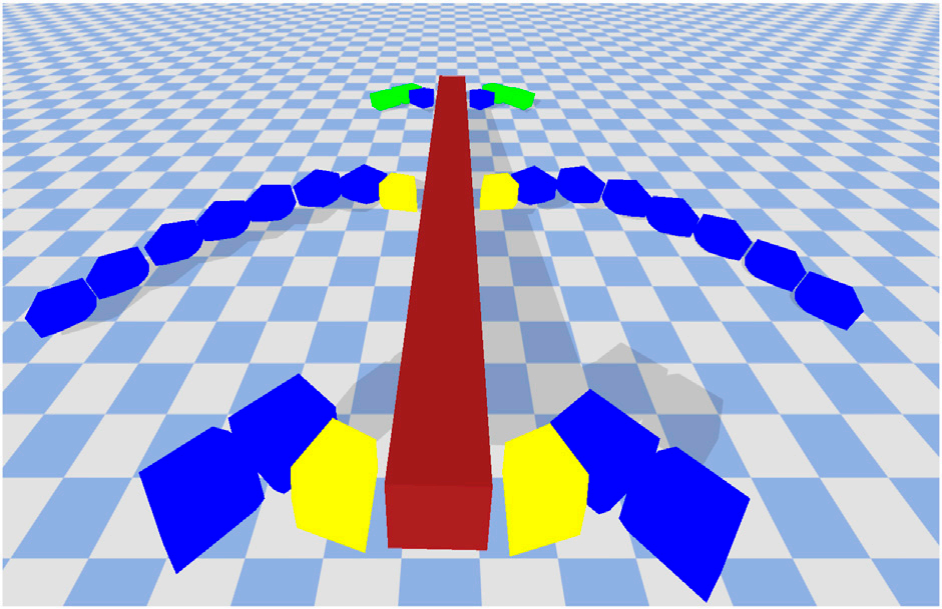
Best performing solution on the high friction surface. Covers 102.22 units in the *x*-direction. Normalized Friction = 0.57, Normalized Restitution = 0.57, Normalized Average Leg Size = 0.47.

**FIGURE 10:**
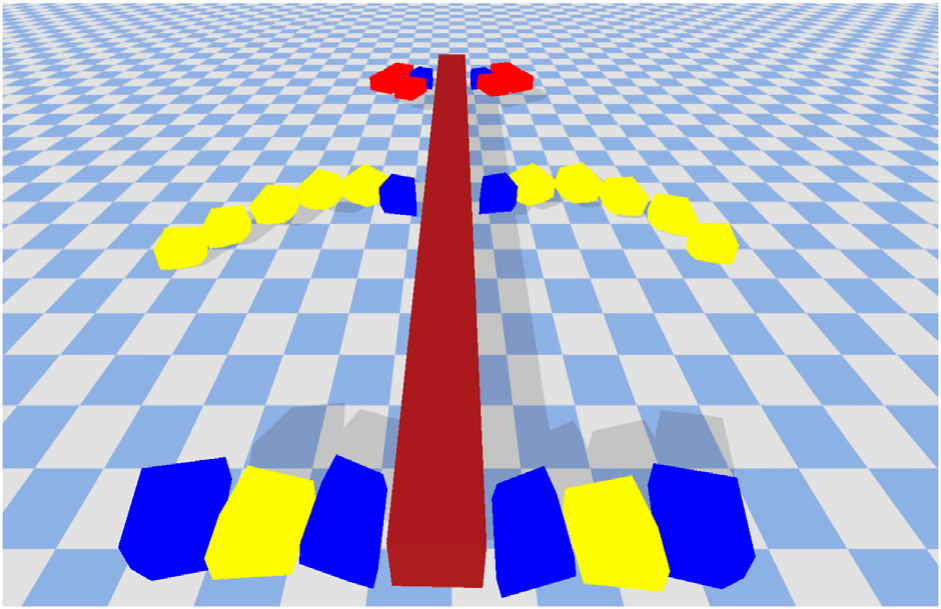
An example of a solution which transfers well to a different environment. This solution had a fitness of 42.14 on the high friction surface and when transferred to the low friction surface it was able maintain a reasonable fitness value of 36.14. Normalized Friction = 0.71, Normalized Restitution = 0.35, Normalize Average Leg Size = 0.41.

We pick two solutions for further analysis. These two are given in Figures 9, 10. The colors denote friction values. Green = 0.25, Red = 0.5, Blue = 0.75, and Yellow = 1.0. The cross-section (maroon) is always given a default friction value of 0.5. The robot in Figure 9 is the best performing solution on the high friction surface. The movement of this robot is mainly guided by the two middle legs. They lift the cross-section off the floor and create forward thrusting motion to move the robot forward. The legs in the middle are mostly made up of high friction building blocks (blue blocks with friction coefficient of 0.75), which when combined with the control parameters allow the robot to make a good grip with the surface without much resistance. The leg movements are fast, which allow the robot to cover quite a lot of distance in a short amount of time. This same robot when transferred to the low friction surface performs very poorly. The legs are not able to create a proper grip with the surface which causes it to create rotating side-ways motion. As mentioned earlier, if a robot rotates beyond a certain threshold, we immediately stop the simulation and assign fitness of 0. This robot ends up suffering from this exact same problem and hence is assigned fitness of 0 on the low friction surface.

The robot in Figure 10 has a fitness value of 42.14 on the high friction surface and 36.14 on the low friction surface. It performs reasonably well on both surfaces. Just like the previous robot, it uses the pair of legs in the middle to create forward thrusting motion in order to move forward. The motion however is much slower and well coordinated. This maybe because the middle legs have the highest frictional properties (yellow blocks with friction coefficient of 1.0) and the resistance from the ground can have a slowing down effect. This solution when transferred to the low friction environment out-performs all other solutions within the final archive. The long high friction legs are able to make a better grip with the surface and utilize the (albeit limited) resistance to make steady forward movements.

In summary, it is apparent that the archive contains solutions which perform well on both environments despite only explicitly being training on one. It may be that some of the emergent behaviors are universal in nature and transfer well across environments. Even though the environments are different in terms of material properties, they are also similar in the sense that they are both flat terrains. Designs which are able to perform reasonably well across both environments are able to model their behavior well for a flat terrain. The inherent diversity of the archive also helps since it maintains designs with wide ranging characteristics that may be useful in alternate environments.

#### 4.2.4 Comparison With Manual Design

**FIGURE 11:**
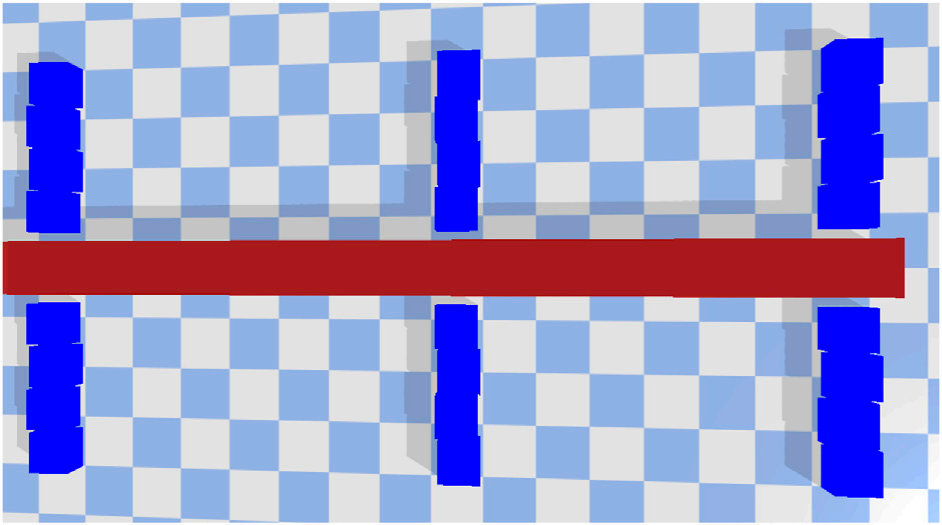
Baseline design. Legs are constructed using four cubes with each having upper-mid-level material properties (friction = 0.75, restitution = 0.75).

We also wanted to demonstrate how a manually designed robot would compare against the designs discovered by the multi-level optimization. This would involve designing all the components manually except for the controller which would be optimized using the procedure outlined in Section 3.4.1. Here we consider a robot (Figure 11) with three legs on each side. The legs are constructed using four square polygons and are created using upper-mid-level material properties (Friction = 0.75 and Restitution = 0.75). The cross-section is 16 units in length. When evaluated under the same environmental conditions, this robot performs poorly in both environments. It covers 2.75 units in the high friction environment and only 0.22 units in the low friction environment. This shows that designing a robot with so many design variables is definitely non-trivial. It also shows the efficiency of our proposed shape grammar and material selection approach. Optimizing and connecting irregular polygons at different angles helps produce better movement. Having legs made up of a combination of material properties also helps.

### 4.3 Limitations

The hierarchical design paradigm presented in this paper has a number of limitations. Firstly, the control system is fairly simplistic. It does not accept environmental input such as sensor data or movement direction (Gangapurwala et al., 2020), and the overall function itself is fairly basic. We are aware that this may impose certain limitations on the complexity of the evolved behaviours. However, we wanted to keep all other components as simple as possible in order to keep the focus of the study on the multi-level architecture and its perceived benefits. In future research we will be using more sophisticated control strategies combined with meaningful sensor data.

The materials library used in this research is also fairly basic in that it only considers two mechanical properties i.e. friction and restitution. In reality, materials are characterized by many different features. The main reason for this simplistic approach is the inherent limitations with robotic simulators in not being able to accurately model physical materials. In future research we will consider using co-simulation and data driven modelling to improve the accuracy with regards to materials. In any case, our proposed hierarchical optimization strategy clearly demonstrates how physical materials can be effectively included in the design process.

Despite these limitations we strongly believe that the research in this paper makes a significant contribution to the evolutionary robotics literature. The MLE framework provides a comprehensive library of evolved components and overall robot morphologies, giving the decision maker a wide range of options to choose from. The legs discovered at the components layer can be reused when searching for optimal robot designs across different environments. This would save considerable computation time. Other benefits with relation to self-optimization and cross environmental applicability have already been detailed above.

## 5 Conclusion

In this paper we presented a bottom-up hierarchical approach to robotic design. A library of materials characterized by friction and restitution were presented at the materials layer. At the components layer, these materials were combined with shape grammar to form robot legs of varying complexity and size. Finally at the robot layer, the legs were combined into a body plan to form complete robots.

These evolved designed were tested on two different environments characterized by varying levels of friction and restitution. The experimental results demonstrate two key aspects. First is the self-optimizing nature of the MLE architecture in which we noted noticeable improvements in fitness at the robot layer as the evolution in the components layer was allocated more and more generations of computational budget. This was particularly visible in the later generations where component libraries built using 1,000 generations allowed the robot layer to reach comparatively higher fitness values as opposed to those which were given lower computational budgets. Next is the cross environment applicability of the evolved designs. When the evolved libraries were transferred to a different environment, some designs performed worse while others performed really well. This indicates that one may only need to optimize for one environment and because of the inherent diversity of the archive, it may already contain solutions which perform well in a different environment.

In future research, we will consider more complex environments and tasks for our robots to further test the usefulness of our hierarchical design paradigm. Physical instantiation of the evolved designs is also something worth looking into. Finally, we will also look into a dynamic materials layer which can actively search for useful materials instead of relying on pre-loaded data.

## Data Availability

The raw data supporting the conclusion of this article will be made available by the authors, without undue reservation.

## Footnotes

1
https://bitbucket.csiro.au/scm/cha778/mle.git
